# Acute effect of low-intensity aerobic exercise on eliciting enhanced parietal activation and promoting executive function performance more than moderate-intensity exercise

**DOI:** 10.3389/fphys.2025.1581481

**Published:** 2025-08-18

**Authors:** Shen Lingling, Chen Huaqing, Li Xuan, Cai Jichen, Li Chenxi, Wang Chuhuai

**Affiliations:** ^1^ Department of Rehabilitation Medicine, The First Affiliated Hospital of Sun Yat-sen University, Guangzhou, China; ^2^ Department of Rehabilitation Medicine, Shenzhen Qianhai Taikang Hospital, Shenzhen, China; ^3^ Key Laboratory of Education Ministry for Modern Design & Rotor-Bearing System, Xi’an Jiaotong University, Xi’an, China

**Keywords:** cognitive benefit prediction, exercise intensity, parietal-cortical activation, motor control, young adults

## Abstract

**Introduction:**

Aerobic exercise intensity differentially impacts cognitive function and brain activity, but the optimal intensity for enhancing cognitive function and cortical activity remains unclear. This study investigates the effects of low- and moderate-intensity aerobic exercise (LAE/MAE) on cognition, gait, and brain dynamics in healthy young adults.

**Methods:**

Forty-nine participants were assigned to stretching (SE), LAE, or MAE groups, and their cognitive function was assessed using various tasks before and after exercise, cortical activation was monitored using functional near-infrared spectroscopy, and gait parameters and stability indices were measured using a video motion and posture analysis system.

**Results:**

The LAE group exhibited significantly improved Stroop task reaction time and reduced deoxyhemoglobin concentrations in key cortical regions (left/right S1, left Broca’s area, and right dorsolateral prefrontal cortex). Greater stride length during aerobic exercise correlated with the Stroop task reaction time, and functional connectivity indices during exercise predicted post-exercise cognitive benefits. Notably, LAE enhanced functional connectivity within the parietal cortex, fostering interconnections between left M1 and nearby brain regions including left S1, right M1, and Wernicke’s area.

**Discussion:**

LAE optimizes parietal functional connectivity and executive speed, with stride length and cortical functional connectivity predicting post-exercise cognitive benefits. These findings advance our understanding of the relationships between exercise and brain health, particularly those linked with motor learning and M1 plasticity-mediated cortical network dynamics.

## Introduction

Exercise has been empirically validated to confer substantial benefits across all organ systems. These benefits encompass enhanced cardiometabolic function, improved motor performance, optimized neuronal activity, and strengthened mental and cognitive faculties. Furthermore, such advantages are observed across diverse populations, including adolescents, middle-aged adults, older persons, and individuals with pre-existing conditions. Ultimately, these multifaceted benefits collectively contribute to improved overall health status and reduced mortality risk (Angevaren et al., 2008; Billinger et al., 2014; Chekroud et al., 2018; Dauwan et al., 2021; Tucker et al., 2022).

Therefore, a key research goal is to quantify exercise regimens that help people engage in physical activity and achieve maximum benefits. Among important exercise factors like intensity, frequency, type (aerobic exercise, resistance training, dance, mind-body practices, etc.), volume, and duration, studies consistently show that intensity is the most crucial measure. It has become the primary indicator for evaluating exercise effectiveness and health benefits. While the 2020 World Health Organization guidelines recommend 150–300 min/week of moderate-intensity or 75–150 min/week of vigorous-intensity aerobic physical activity (Bull et al., 2020), emerging evidence confirms minimal exercise regimens can elicit meaningful effect: brief (4-s) bursts of activity improve muscle mass and aerobic power (Allen et al., 2021); 7,000 daily steps reduce all-cause mortality (Lee et al., 2019; Saint-Maurice et al., 2020); low-intensity walking preserves white matter integrity (T1W/T2W signal changes linked to episodic memory improvement) (Chen et al., 2020; Mendez Colmenares et al., 2021); resistance training upgrades executive function scores on Stroop test (Chow et al., 2021); yoga decreases anxiety symptoms (Cole et al., 2022); and Tai Chi exhibits clinically significant cognitive gains (like MoCA, MMSE, Flanker test and so on) (Liu et al., 2021). Notably, neuroimaging data suggest these behavioral changes may coincide with structural/functional neural reorganization, though causal mechanisms require further verification. For cognitive benefit, the optimal exercise intensity remains disputed across studies involving participants of varying ages, although moderate-intensity aerobic exercise is consistently the universally advocated strategy (Yanagisawa et al., 2010; Sanders et al., 2020; Fernandez-Rodriguez et al., 2022; Gallardo-Gomez et al., 2022; Yu et al., 2022; Mao et al., 2023). A recent systematic review and meta-analysis (Hortobagyi et al., 2022) explored how exercise intensity impacts three key areas: (1) Motor-neuroplastic coupling: both low- (<77% of maximal heart rate [HRmax]) and high-intensity exercise (>77% of HRmax) enhanced neuroplasticity (functional activation, brain structure and neurochemicals), correlating with improved motor performance (strength, gait, and balance metrics); (2) Cognitive effects: no discernible associations were observed between these neuroplasticity enhancements and cognitive outcomes (executive function, memory assessments, and processing speed); (3) Population specificity: intensity-neuroplasticity correlations were significant in healthy young adults, but absent in healthy older adults or neurological patient. These findings highlight critical gaps in current biomarkers’ capacity to explain exercise-induced cognitive benefits, underscoring the need for mechanistic studies comparing intensity effects through multimodal neural signatures.

To address the biomarker gaps identified, current research converges on three complementary domains: (1) Established markers: brain-derived neurotrophic factor (BDNF) shows strong correlations with exercise-induced cognitive benefits across ages and species (humans/rodents), persisting across exercise modalities and intensities. However, these associations are is not always observed, and the changes in BDNF were not consistent with cognitive performance (Choi et al., 2018; Gaitan et al., 2021; Ruiz-Gonzalez et al., 2021; Chan et al., 2022; Guo Yi et al., 2025); (2) Novel candidates: Preliminary studies identify potential biomarkers with inconsistent correlations: (a) Molecular: insulin-like growth factor, skeletal muscle mitochondrial metabolites, and neuroinflammation factors (Pedersen, 2019; Gaitan et al., 2021; Stein et al., 2021); (b) Physiological: gait efficiency (stride length, speed, and daily step count), balance capabilities, and cardiorespiratory fitness (Montero-Odasso et al., 2017; Intzandt et al., 2018; Sanders et al., 2020; Liu et al., 2023; Wang et al., 2023); (3) Neuroimaging signatures: Neuroimaging provides evidence of brain changes after exercise, but cannot draw causal conclusions: (a) Functional: Cerebral blood flow, brain glucose metabolism, electroencephalographic, and network connectivity dynamics; (b) Structural: frontal-parietal and hippocampal areas reorganization (Chu et al., 2017; Stern et al., 2019; Guadagni et al., 2020; Zhu et al., 2021; Lenze et al., 2022; Tarumi et al., 2022; Kuwamizu et al., 2023). Thus, establishing reliable biomarkers remains critical to enhance precision in exercise prescription and adherence, particularly given exercise’s role as a cost-effective intervention requiring sustained engagement.

Functional near-infrared spectroscopy (fNIRS) is an optical neuroimaging technique that detects neural activity through real-time monitoring of oxygenated hemoglobin (HbO) and deoxygenated hemoglobin (HbR) concentration dynamics. Unlike various other biological markers, it offers advantages such as noninvasiveness, moderate temporal and spatial resolution, enhanced resistance to motion artifacts, ease of operation, and cost-effectiveness, which render it highly suitable for scientific research pertaining to exercise and cognition (Herold et al., 2018). Aerobic exercise (comprising walking, running, cycling, etc.) is the main exercise used in fNIRS studies to enhance cognitive function. However, few studies measure brain activity during actual exercise. Most focus on testing brain activity pre- and post-exercise during cognitive tasks. A limited number of studies have used fNIRS to capture brain activity during dual-task paradigms involving cycling exercise coupled with cognitive tests (e.g., Stroop task, n-back task) or walking coupled with cognitive tests (e.g., two-back task) (Huang et al., 2019; Tempest and Reiss, 2019; Talamonti et al., 2022; Zheng et al., 2022). In these studies, elucidating the mechanisms by which exercise improves cognition has been challenging, and the studies have failed to provide a robust indicator capable of predicting the cognitive benefits of exercise.

To bridge these methodological gaps in real-time neural monitoring and predictive biomarker identification, we used 63-channel fNIRS technology, which encompasses crucial brain regions such as the frontal, parietal, and occipital lobes, to continuously monitor alterations in cerebral cortical oxygenation levels during exercise. This protocol achieves three possibilities: (1) Dynamic brain mapping through continuous cortical oxygenation tracking during active exercise; (2) Network neuroscience analysis of frontal-parietal-occipital functional connectivity; (3) Predictive modeling integrating exercise induced cognitive benefit with cerebral hemodynamics, gait analytics and cardiopulmonary. We hypothesized that aerobic exercise could improve cognitive function by increasing brain functional connectivity and activating cognitive-related brain regions such as the prefrontal, parietal lobes and so on. Moreover, fNIRS, gait, and cardiopulmonary function indices may serve as predictive indicators of cognitive benefits. By integrating pre- and post-exercise cognitive function assessments with concurrent monitoring of physiological parameters during exercise (including cardiorespiratory function, exercise intensity, and gait characteristics), we comprehensively analyzed the relationships between these factors and the cognitive benefits induced by exercise. Our goal was to identify robust biological predictors for and reveal the neural mechanisms underlying exercise-mediated enhancements in cognitive function. Ultimately, this study provides a scientific basis for developing personalized cognitive intervention strategies. Furthermore, our findings hold major implications for promoting public health, preventing cognitive decline, and enhancing the quality of life among diverse populations.

## Materials and methods

### Participants

Fifty-eight healthy adults were recruited in this study. The inclusion criteria were (1) age between 20 and 40 years, (2) absence of chronic conditions that could potentially affect the experimental outcomes, and (3) right-handedness. The exclusion criteria were (1) functional impairments that precluded the completion of cognitive tests; (2) a history of medication usage within the past 6 months that might have influenced cognitive functions; (3) uncontrolled hypertension, ischemic cardiomyopathy, malignant arrhythmia, lower limb pain, or other chronic conditions impeding physical activities; and (4) involvement in any other clinical trials. The withdrawal criteria were (1) inability of the participant to complete the trial due to reasons such as arrhythmia, uncontrollable hypertension, lower limb pain, muscle strain, inability to achieve the target heart rate, inability to comprehend the cognitive test instructions, or any other factors that hindered trial completion; and (2) participant-initiated withdrawal, including but not limited to intolerance to exercise intervention, inability to complete cognitive tests, or voluntary withdrawal without specific reasons. This research was approved by the Clinical Research and Experimental Animal Ethics Committee of the First Affiliated Hospital of Sun Yat-sen University and adhered to the latest version of the Helsinki Declaration ([2024]469), and was registered on medicalresearch.org (MR-44-24-051877). Written informed consent was obtained from all participants. Table 1 depicts the characteristics of the participants.

**TABLE 1:** Demographics and baseline clinical characteristics of all participants.

Variables	Groups	Control exercise Stretching exercise (n = 17)	Aerobic exercise		*p* value
			Low-intensity (n = 15)	Moderate-intensity (n = 17)	
Sex	Male	6/35.3%	5/33.3%	6/35.3%	0.991
	Female	11/64.7%	10/66.7%	11/64.7%	
Age (years)		25.76 ± 3.527	24.80 ± 3.212	24.88 ± 4.581	0.727
Dominant hand (right)		17/100%	15/100%	17/100%	—
Marriage	Unmarried	15/88.2%	15/100%	17/100%	0.141
	Married	2/11.8%	0/0%	0/0%	
Education (years)		17.71 ± 1.448	17.07 ± 2.282	17.06 ± 2.106	0.555
Occupation	Full-time job	17/100%	14/93.3%	16/94.1%	0.380
	Part-time job	0/0%	0/0%	1/5.9%	
	Unemployed	0/0%	1/6.7%	0/0%	
Social interaction	High	8/47.1%	5/33.3%	2/11.8%	0.080
	Low	9/52.9%	10/66.7%	15/88.2%	
Height (m)		1.6888 ± 0.08609	1.6567 ± 0.08217	1.6382 ± 0.07222	0.189
Weight (kg)		62.71 ± 10.276	60.67 ± 10.544	59.21 ± 10.409	0.619
BMI		21.854 ± 1.908	21.979 ± 2.397	21.931 ± 2.595	0.988
Heart rate		79.88 ± 9.610	79.87 ± 14.638	78.65 ± 6.698	0.929
Blood pressure	SBP	114.76 ± 12.075	114.67 ± 12.286	110.00 ± 11.570	0.429
(mmHg)	DBP	77.76 ± 9.679	75.80 ± 7.839	74.88 ± 6.051	0.791
Past medical history	No chronic diseases	17/100%	15/100%	17/100%	—
	No medication	17/100%	15/100%	17/100%	—
Regular exercise	Non-being	9/52.9%	8/53.3%	8/47.1%	0.921
	Being	8/47.1%	7/46.7%	9/52.9%	
Exercise duration (min/week)		231.18 ± 303.498	144.00 ± 193.494	187.06 ± 195.640	0.589
Daily step count		7,205 ± 2,942	8,292 ± 2,581	9,705 ± 3,674	0.075

The results of numerical variables are presented as means ± standard deviation. The comparison of means between the three groups is performed using one-Way ANOVA., The results of categorical variables are presented as frequency (n) and percentage (%), and the comparison of frequency between the three groups is performed using the Chi-Square test. BMI, body mass index; SBP, systolic blood pressure; DBP, diastolic blood pressure.

### Experimental design

**FIGURE 1:**
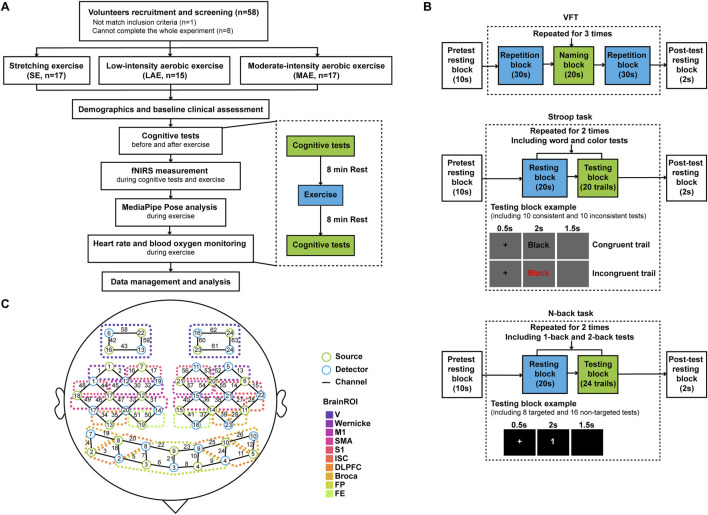
Experimental design and methodology. **(A)** Flow-chart through this study. **(B)** Experimental paradigm of cognitive tests including VFT, Stroop task and n-back task. **(C)** The distribution of 63 fNIRS channels in frontal, parietal and occipital lobes. BrainROI: the brain regions of interest; V: Visual cortex; M1: Primary motor cortex; SMA: Pre-motor and supplementary motor cortex; S1: Primary somatosensory cortex; lSC: left subcentral area; DLPFC: Dorsolateral prefrontal cortex; FP: Frontopolar area; FE: Includes frontal eye fields.

This study initially screened 58 participants. One individual was excluded due to congenital heart conditions, and 8 withdrew because of intolerance to the acute aerobic exercise protocol or scheduling conflicts preventing completion. Ultimately, 49 participants ultimately selected were randomly assigned to three groups, the stretching exercise group (SE group, n = 17), low-intensity aerobic exercise group (LAE group, n = 15), and moderate-intensity aerobic exercise group (MAE group, n = 17), and then the following tests and evaluations were conducted sequentially (Figure 1A): (1) Demographic data collection: age, sex, height, weight, education level, dominant hand, social status, and physical activity levels over the past 6 months (regular exercise habits, exercise duration per week and daily walking steps). (2) Pre-trial physical capacity assessment: baseline heart rate, blood pressure, and respiratory measurements. Participants were also queried regarding their history of chronic diseases, medication use, and smoking and alcohol consumption habits. (3) Baseline cognitive function assessment: Participants wore a 63-channel fNIRS device while performing three cognitive tests, the verbal fluency test (VFT), Stroop task, and n-back task. (4) Exercise intervention: After completing the cognitive tests, participants rested for 8 min (as previously reported (Herold et al., 2018), this duration allows brain activity to return to baseline levels). Subsequently, the exercise interventions were administered: the aerobic exercise groups performed low-intensity aerobic exercise or moderate-intensity aerobic walking on a treadmill, whereas the SE group engaged in stretching exercises targeting the head, neck, and torso on a treadmill. During the intervention, fNIRS was used to capture changes in cerebral cortical oxygenation reflecting brain activity, heart rate monitors were employed to record heart rate and blood oxygen saturation, and a dual-channel camera coupled with a MediaPipe Pose system was used to analyze gait and pose metrics. (5) Post-exercise cognitive function assessment: After an 8-min rest period, participants were asked whether they had returned to their baseline state. Prior to donning the 63-channel fNIRS device, heart rate was re-measured to ensure it had returned to individual baseline levels. Participants then repeated the three cognitive tasks: VFT, Stroop task, and n-back task.

### Exercise procedure

All participants were instructed to refrain from vigorous activity for 24 h and avoid caffeinated beverages (e.g., coffee, tea) on the testing day. Testing occurred under rigorously standardized conditions: wearing athletic footwear and clothing, using the same calibrated motorized treadmill in a controlled laboratory environment (e.g., lighting, spatial context).

We employed the percentage of maximum heart rate (HRmax) as the target heart rate (THR) to define exercise intensity. Participants in the aerobic exercise groups were instructed to perform aerobic walking on a treadmill, aiming to attain the THR within 5 min and then maintain this THR range through continuous aerobic walking for 10 min. Subsequently, a 5-min cool-down period was implemented, during which the walking speed and heart rate were gradually reduced to achieve a resting state. Conversely, participants in the SE group engaged in a 10-min stretching routine targeting the head, neck, and torso, guided by a video. In the SE group, the treadmill was strictly utilized as a static, zero inclined platform to standardize environmental conditions (e.g., lighting, spatial context) and upright posture across all experimental groups. No specific THR was required for this group, and a 5-min marching exercise was performed both before and after the stretching session. HRmax was defined as 220 minus current age (in years), with the THR for moderate-intensity and low-intensity exercise set at 50%–70% and <50% of HRmax, respectively, according to American College of Sports Medicine (ACSM) recommendations. Given the time-intensive protocol and resource constraints, we prioritized non-invasive methodologies over direct lactate measurement or maximal oxygen uptake (VO_2_max) assessment via cardiopulmonary exercise testing (CPET). Target heart rate (%HRmax) was selected as the primary intensity metric based on: (1) Strong validation: In healthy young adults (excluding athletes/obese individuals), %HRmax demonstrates high correlation with VO_2_max (r = 0.87-0.89) (Lounana et al., 2007; Nikolaidis, 2014). Specifically, 40%, 60%, 80%, and 85% VO_2_max correspond to 63%, 76%, 89%, and 92% HRmax respectively (Swain et al., 1994); (2) Population-specific accuracy: The Fox formula (HRmax = 220 − age) is recommended for use in common, young, healthy adults (Tanaka et al., 2001; Nikolaidis, 2014; Nikolaidis et al., 2018), and shows clinically acceptable agreement with CPET-measured values (Villafaina et al., 2022); (3) Operational feasibility: Avoids exercise disruption from invasive procedures (e.g., blood sampling) while enabling real-time intensity modulation.

### Cognitive tests

Before and after exercise, the VFT, Stroop task, and n-back task were sequentially administered to assess the participants’ verbal abilities, executive functions, and working memory, respectively. The experimental paradigm is illustrated in Figure 1B.

### VFT

The VFT comprised two fixedly sequenced components: two repetition tasks and three naming tasks. In the repetition tasks, participants were instructed to recite the sequence “1, 2, 3, 4, 5” within 30 s. In the naming tasks, the participants were required to swiftly utter as many words as possible from a specified categorical concept within 20 s. For instance, upon hearing the cue “animals,” participants were expected to verbalize “cat,” “elephant,” etc. The number of words spoken and their accuracy were meticulously recorded for subsequent analysis.

### Stroop task

The Stroop task is composed of two modules, the word test and the color test, each containing 40 trials (20 congruent and 20 incongruent trials). During each trial, a “+” symbol is displayed at the center of a computer screen for 0.5 s, followed by a stimulus for 2 s and a 1.5-s blank interval. Participants are instructed to respond within the subsequent 2 s to the stimulus, which is a word (color word) displayed in one of five colors (red, yellow, green, blue, or black). In the congruent condition, the center of the screen shows a color word whose meaning matches the displayed color; by contrast, in the incongruent condition, a color word is shown with its printed color differing from the meaning of the word (e.g., “Black” printed in red). These two conditions are presented in a randomized order. In the word test, participants press a corresponding key based on the semantic meaning of the color word, whereas in the color test, they respond by pressing a button that corresponds to the color of the color word. The number of correct responses, the reaction times, and the ratio of reaction times between congruent and incongruent conditions are recorded. A 20-s resting block is provided before every 20 trials.

### N-back task

The n-back task encompasses two modules, 1-back and 2-back, each consisting of 24 trials (8 target trials and 16 non-target trials, corrected from the original) presented in a randomized order, with the entire sequence repeated once. Within each trial, a “+” symbol is displayed at the center of a computer screen for 0.5 s, followed by a digit (stimulus) for 2 s, and a subsequent 1.5-s blank interval. Participants are required to respond within 2 s to the stimulus by pressing the left button (√) if the stimulus matches (i.e., if the presented digit is identical to the one n steps back in the sequence) and the right button (×) if it does not match. Initial practice sessions (8 trials each) are conducted for both the 1-back and 2-back tasks to ensure comprehension by the participants. The number of correct responses and response times are recorded. A 20-s rest period is provided before each module.

### fNIRS data acquisition and processing

A 63-channel fNIRS system (NirSmartII-500pro, Danyang Huichuang Medical Equipment Co., Ltd., Jiangsu, China) was used to capture dynamic alterations in the concentrations of oxygenated hemoglobin (HbO) and deoxygenated hemoglobin (HbR) within the frontal, parietal, and occipital lobes of the brain. The elasticated fNIRS headcap maintained optode positioning with minimal displacement during movement, as confirmed by participant feedback and post-session channel quality checks. Operating at wavelengths of 730 and 850 nm, the system acquired data at a sampling rate of 11 Hz. A total of 63 channels, each defined as the midpoint between a paired light source and detector, were established, using 24 light sources and 24 detectors strategically positioned in accordance with the standard international 10–20 system of electrode placement. Specifically, D21 was positioned at C3, S17 at C4, and D3 at Fpz (Figure 1C). To precisely map probe positions (conforming to the 10–20 system: Nz, Cz, Al, Ar, Iz), an electromagnetic 3D digitizer (Patriot, Polhemus, VT, United States) was used on a standardized head model. Subsequently, the grand-averaged coordinates were processed in NirSpace software (Danyang Huichuang Medical Equipment Co., Ltd.), enabling the estimation of Montreal Neurological Institute (MNI) coordinates. These MNI coordinates, stemming from Monte Carlo simulations modeling light pathways, were integrated with 3D positioning and mapping techniques to ascertain channel spatial localization. Each MNI coordinate served as the centroid of a 10 mm radius sphere. Within this sphere, the overlap probability of individual brain regions was quantified by calculating the ratio of the voxel count of each brain region covered in this sphere to the total voxels in the sphere (Lancaster et al., 2007; Tsuzuki et al., 2007; Laird et al., 2010). Because of the nature of near-infrared technology, the MNI coordinate might not align exactly with the center of brain regions. Through analysis, we identified the brain region with the highest probability (prefer ≥50%) under each fNIRS channel and defined the region of interest (ROI) accordingly (Figure 1C; Supplementary Table S1). Furthermore, by implementing a right-hand grip task, we confirmed significant activation in the left primary motor cortex (M1) detected by fNIRS, thereby further validating the accuracy of our spatial localization.

The fNIRS data were preprocessed using NirSpark software (Danyang Huichuang Medical Equipment Co., Ltd.). First, the raw fNIRS signal was converted into changes in optical density by taking the logarithm of the signal. Second, to mitigate the influence of instrumental noise and physiological artifacts (breathe, heartbeat), bandpass filtering was employed, with a high-pass filter set at 0.01 Hz and a low-pass filter set at 0.2 Hz. Third, alterations in HbO and HbR concentrations were derived using the modified Beer-Lambert law. In the context of cognitive testing, the block-average values of HbO and HbR during the Stroop task were computed. For the exercise procedure, functional connectivity analysis within the brain network, specifically between channels and ROIs, during the 10-min sustained exercise period was performed for both HbO and HbR by using Pearson’s correlation coefficient. This comprehensive data processing methodology was adopted in accordance with previous studies (Zhang et al., 2021; Li et al., 2023; Yan et al., 2024). To present the connectivity heatmap, we used an online plotting tool available at https://www.chiplot.online/. To mitigate the potential for a low proportion of false positives, we implemented a rigorous statistical thresholding approach, setting the q-value to be < 0.05, with false-discovery-rate (FDR) correction applied.

### Heart rate and blood oxygen monitoring

Heart rate and blood oxygen saturation were monitored in real time using a wearable, wireless sensor device (Fengling Technology Co., Ltd., Dongguan, China), manufactured with two LEDs emitting at 660 and 880 nm, that operates continuously for 2 h at 2.4 GHz and enables seamless data transmission up to 5 m for remote monitoring of heart rate and blood oxygen saturation. Furthermore, we have developed a dedicated data processing program, which employs the widely recognized photoplethysmogram (PPG) technique (Tang et al., 2009), to rapidly and accurately calculate heart rate and blood oxygen saturation values on a computer platform. This program, which represents a major advance in the realm of physiological monitoring, allows researchers and clinicians to perform real-time analysis of critical vital signs.

### Gait and pose analysis

For real-time body pose tracking, we used MediaPipe BlazePose, a cross-platform framework featuring a comprehensive suite of sophisticated tools that efficiently translate physical phenomena into digital information and thus provide exhaustive solutions for sign language recognition, facial identification, and posture estimation. By employing the MediaPipe Pose Landmarker task, we can accurately extract anatomical landmarks from both still images and video sequences depicting human bodies (Lugaresi et al., 2019). The validation results, including the 95% limits of agreement and mean differences between MediaPipe and reference devices (a clinical goniometer, Angle Ruler 50, and ADA 360° digital protractor), demonstrate that MediaPipe exhibits high reliability and validity (Latreche et al., 2023).

**FIGURE 2:**
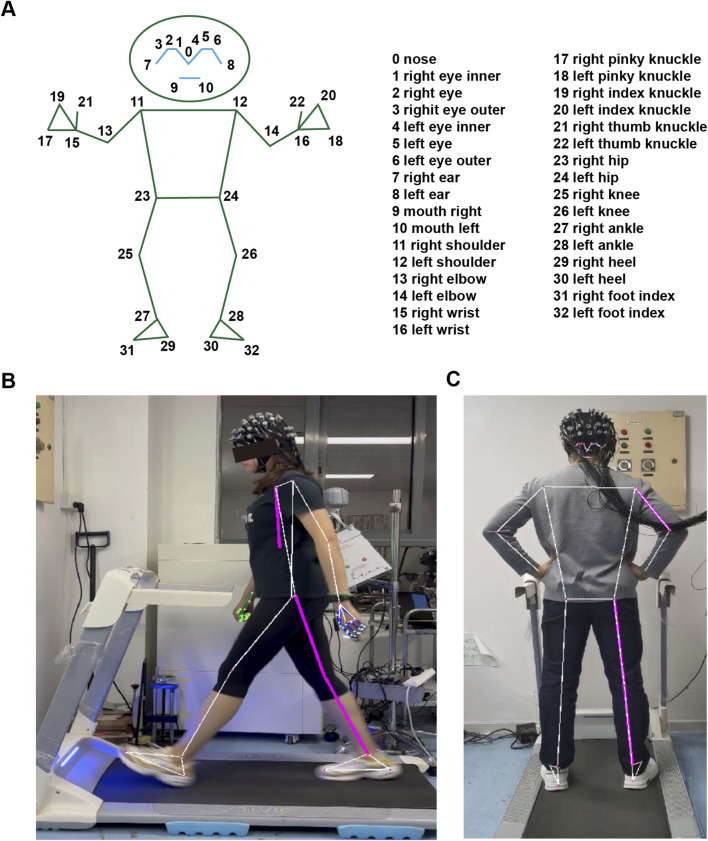
MediaPipe BlazePose system for gait and pose analysis. **(A)** Schematic diagram of 33 pose landmarks. **(B,C)** Demonstration of the two-stage detector-tracker approach for pose estimation. **(B)** Displays the left-field view, and **(C)** presents the back-field view, highlighting the accurate detection of pose landmarks in a dynamic environment.

In this study, we used MediaPipe BlazePose with a two-stage detector-tracker approach for pose estimation (Fengling Technology Co., Ltd.). Here, the tracker initially processes the input image to pinpoint the ROI where the human pose resides, and then within this designated ROI, pose key points are precisely identified. When processing video files, the detector initially identifies the ROI in the first frame, and for subsequent frames, the ROI is dynamically updated based on the pose key points detected in the preceding frame, as elaborated further in subsequent sections. The task culminates in the estimation of 33 pose landmarks, each of which is annotated with both normalized and world coordinates (Figure 2). These landmarks serve as the foundation for swiftly computing joint angles and angular velocities, thereby enabling in-depth analyses of body posture, identification of critical positions, and classification of human actions. Furthermore, by integrating user height data, the analysis can be extended to calculate joint motion displacement, which then facilitates real-time center of gravity estimation and comprehensive walking gait analysis.

### Sample size calculation

Based on the analysis of our previous pilot study, PASS software (2021) was used to estimate the sample size. Based on reaction time in the Stroop task pre- and post-exercise, input parameters included group-specific means, between-groups mean square and with-subjects mean square error derived from pilot data (n = 4, two-way ANOVA). With an α level of 0.05, and a power of 0.80, we determined that a minimum of 45 patients was required. To accommodate a possible dropout rate of 20%, we should enroll a total of 54 participants for this study.

### Randomization and blinding

Randomization was performed using a computer-generated random sequence Microsoft Excel (2019) to allocate eligible participants into three groups in a 1:1:1 ratio. To ensure balanced baseline characteristics, post-hoc stratification adjustments were applied based on demographic and clinical characteristics (e.g., gender, physical history). Due to the interactive nature of the intervention, neither participants nor treating clinicians were blinded to group assignments. All cognitive outcomes were objectively measured (e.g., reaction time, task accuracy) using standardized computerized protocols, eliminating reliance on subjective evaluations. Outcome assessors followed strict data collection guidelines to minimize operator-dependent variability. Data analysts remained blinded to group allocations during statistical processing to ensure unbiased interpretation.

### Statistical analysis

All statistical analyses were performed using SPSS v25.0 (IBM Corp., Armonk, NY, United States). The homogeneity of variances and the normality of the distribution of parameters were tested using Levene’s and Kolmogorov-Smirnov tests, respectively. In the statistical analysis of demographic and clinical characteristics, the results for numerical variables are presented as means ± standard deviation, with the means among the three groups compared using one-way ANOVA. For categorical variables, the results are displayed as frequencies (n) and percentages (%), with the proportions among the three groups compared using the Chi-square (χ^2^) test. For continuous data, values are expressed as means ± standard deviation. To analyze the differences between pre-exercise and post-exercise conditions in three group (SE, LAE and MAE group), a two-way repeated measure ANOVA (time × group) was applied to determine the presence of interaction effects. If a significant interaction appeared, a post hoc analysis with a Sidak adjustment was carried out for multiple comparisons. Multiple groups were compared using one-way ANOVA, accompanied by the Tukey’s multiple comparisons test for data exhibiting normal distribution and the Games-Howell’s multiple comparisons test for data deviating from normal distribution. Lastly, Pearson’s correlation coefficient was used to assess the strength and direction of the relationship between the variables. Differences were considered statistically significant at *p* < 0.05.

## Results

### Demographic and clinical characteristics

This study was completed by all 49 participants, who were randomly classified into SE, LAE, and MAE groups. No statistically significant differences were observed in the characteristics of the participants, including sex, age, dominant hand, marriage, education, occupation, social interaction, height, weight, body mass index, basal heart rate and blood pressure, and exercise habit (*p* > 0.05, Table 1; Supplementary Table S2).

### Cognitive responses

To compare the effect of the three exercise regimens on cognitive function, the participants were asked to perform the VFT, Stroop task, and n-back task pre- and post-exercise. Before conducting the analysis, we rigorously assessed the quality of the data and implemented a stringent exclusion criterion to eliminate any abnormal data points. This included the removal of samples with atypically low accuracy scores (defined as 0% accuracy in isolated task modules coupled with normal performance in other comparable tasks, indicating technical/operational anomalies rather than cognitive deficits), specifically one sample from the SE group and one from the LAE group in both the Stroop task and the n-back task. We also excluded data in the case of participants whose data were lost due to equipment malfunction: one sample from the SE group, two samples from the LAE group, and one sample from the MAE group in the visual feature tracking VFT task. These measures ensured the integrity and reliability of the dataset for subsequent analysis.

**FIGURE 3:**
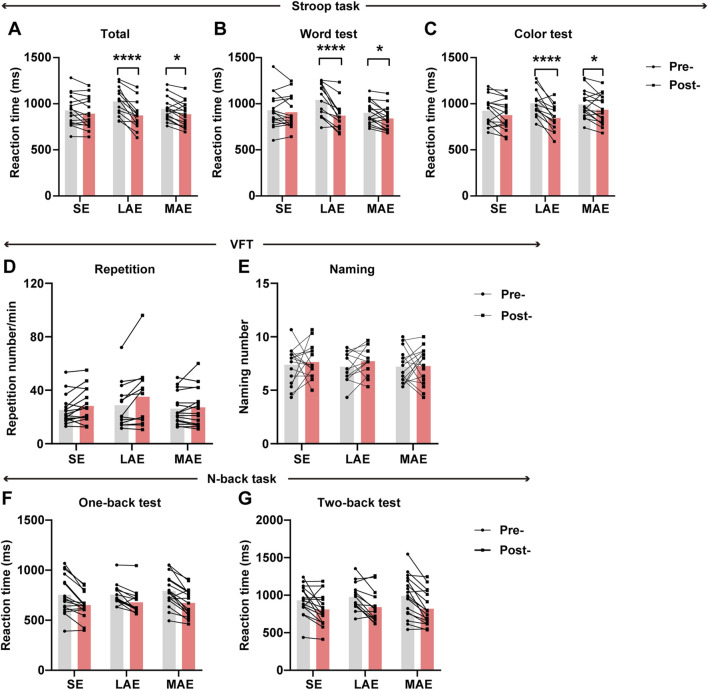
Cognitive performance across different exercise groups. **(A–C)** Change in reaction time (ms) pre- and post-exercise in the Stroop task, Stroop word test and color test. n = 16 for SE group, 14 for LAE group and 17 for MAE group, two-way ANOVA. **(D,E)** Change in reaction time (ms) pre- and post-exercise in the one-back test **(D)** and two-back test **(E)** of n-back task. n = 16 for SE group, 14 for LAE group and 17 for MAE group, two-way ANOVA. **(F,G)** Change in repetition number per minute **(F)** and naming number **(G)** pre- and post-exercise in the VFT. n = 16 for SE group, 14 for LAE group and 17 for MAE group, two-way ANOVA. *p < 0.05, **p < 0.01, ***p < 0.001, ****p < 0.000, error bars indicate SD.

We observed a significant post-exercise improvement in reaction time during the Stroop task in the LAE group, which was less pronounced in the MAE group and absent in the SE group (Figure 3A, F = 8.740, p = 0.0006, η^2^partial(p) = 0.284). And the improvement was evident in both the Stroop word test (Figure 3B, F = 9.834, p = 0.0003, η2 (p) = 0.308) and the color test (Figure 3C, F = 5.884, p = 0.0054, η2 (p) = 0.211). Notably, by comparing the ratio of post-to pre-exercise reaction time in the Stroop task, word test and color test, we found the cognitive benefit of exercise was markedly significant in the LAE group, as compared to the MAE and SE groups (Supplementary Figure S1A–C). Additionally, the comparisons of accuracy are not significant in Stroop task after exercise intervention (Supplementary Table S3), which may reflect a ceiling effect in healthy young adults. Collectively, these results indicate that low-intensity aerobic exercise exerts an expediting influence on reaction time in both the word and color components of the Stroop task. However, this beneficial effect appears to be attenuated in the case of moderate-intensity aerobic exercise, highlighting the differential impact of exercise intensity on cognitive processing.

However, there was no significant time × group interaction effect in the repetition test and naming test of the VFT task (Figure 3D, F = 2.530, p = 0.0917, η2 (p) = 0.107; Figure 3E, F = 0.1859, p = 0.8310, η2 (p) = 0.008). In the n-back task, we also didn’t observe significant time × group interaction effect neither in 1-back or in 2-back test (Figure 3F, F = 0.9837, p = 0.3820, η2 (p) = 0.042; Figure 3G, F = 0.4997, p = 0.6101, η2 (p) = 0.022 and Supplementary Table S3).

### Cortical activation during stroop task

**FIGURE 4:**
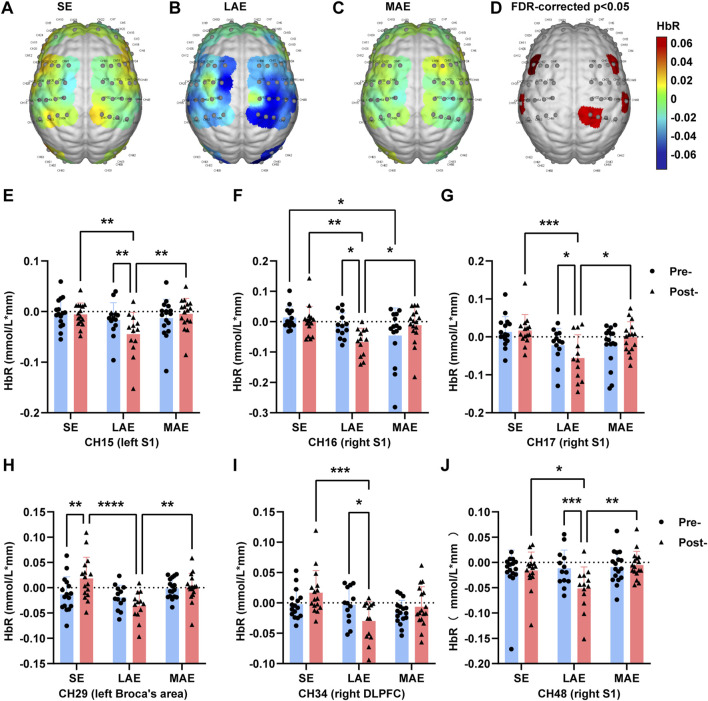
fNIRS activation patterns during Stroop task. **(A–D)** The Brain averaged activation maps of HbR changes for SE group **(A)**, LAE group **(B)** and MAE group **(C)** during Stroop task post-exercise. **(D)** And the statistically significant differences in HbR activation channels among SE, LAE and MAE groups after exercise. n = 16 for SE group, 13 for LAE group and 17 for MAE group, one-way ANOVA with FDR correction applied. **(E–J)** The HbR concentration in channel 15 **(E)**, 16 **(F)**, 17 **(G)**, 29 **(H)**, 34 **(I)**, 48 **(J)** among three groups pre- and post-exercise. n = 16 for SE group, 13 for LAE group and 17 for MAE group, two-way ANOVA. *p < 0.05, **p < 0.01, ***p < 0.001, ****p < 0.000, error bars indicate SD.

Because low-intensity exercise improved executive function, we next examined how exercise affects cortical activation during the Stroop task by using a 63-channel fNIRS system and focusing on the frontal, parietal, and occipital cortex. We excluded three samples from the analysis because of incomplete datasets; specifically, we excluded two samples owing to the absence of cognitive data and one sample due to an equipment malfunction that resulted in a lack of fNIRS data. These exclusions were implemented to ensure the integrity and validity of the remaining data (n = 16 for SE group, 13 for LAE group, and 17 for MAE group) for the subsequent analysis. Notably, our results showed significant differences post-exercise in the averaged HbR concentration of the brain cortex across the three groups in specific channels (15 [left S1], 16 [right S1], 17 [right S1], 29 [left Broca’s area], 34 [right dorsolateral prefrontal cortex, or DLPFC], and 48 [right S1]) (Figures 4A–D); these differences were absent pre-exercise (Supplementary Figure S2A–D). Specifically, a two-way repeated measure ANOVA (time × group) shown that in channels 15, 16, 17 and 48, the HbR concentration was significantly lower in the LAE group post-exercise than in the SE and MAE groups post-exercise and in LAM group pre-exercise (Figures 4E–H,J, CH15: F = 4.275, p = 0.0203, η2 (p) = 0.165; CH16: F = 3.687, p = 0.015, η2 (p) = 0.113; CH17: F = 5.752, p = 0.001, η2 (p) = 0.169; CH48: F = 6.052, p = 0.0048, η2 (p) = 0.219). Furthermore, relative to the SE and MAE group, the LAE group showed significantly reduced HbR concentration in channels 29 post-exercise (Figure 4H, F = 4.275, p = 0.0203, η2 (p) = 0.165), and the HbR concentration in channel 34 in the LAE group post-exercise was significantly lower than in the SE group post-exercise and in LAM group pre-exercise (Figure 4I, F = 4.866, p = 0.0124, η2 (p) = 0.184). These results indicate enhanced cortical activation in certain brain regions, including S1, left Broca’s area, and right DLPFC during the Stroop task following low-intensity aerobic exercise. To further investigate the relationship between the exercise-induced improvement and heightened cortical activation, we conducted Pearson’s correlation analysis on the post-exercise data, examining the correlation between the ratio of post-to pre-exercise reaction time in the Stroop task and the HbR concentration in the aforementioned channels (15, 16, 17, 29, 34, and 48) across the three groups. Only a weak and nonsignificant correlation was detected in channel 16 (Pearson r = 0.2898, *p* = 0.0508), indicating that additional factors might underlie the observed exercise-mediated effects on cortical activation during the Stroop task.

To gain further insight into this matter, we repeated the analysis, this time differentiating between the word and color components of the Stroop task. In the word test of the Stroop task, significant differences were measured in the average HbR concentration among the three groups post-exercise in channels 15, 29, 45 (right S1), and 48 (Supplementary Figure S3A–D), but no such differences were observed pre-exercise (Supplementary Figure S2E–H). Specifically, a two-way repeated measure ANOVA (time × group) shown that in channels 15, 45 and 48, the HbR concentration was significantly lower in the LAE group post-exercise than in the SE and MAE groups post-exercise and in LAE group pre-exercise (Supplementary Figure S3E, G, H, CH15: F = 6.852, p = 0.0026, η2 (p) = 0.241; CH45: F = 5.589, p = 0.007, η2 (p) = 0.206; CH48: F = 9.261, p = 0.0005, η2 (p) = 0.301). And in channel 29, a significantly decrease of HbR concentration was observed in the LAE group relative to the SE and MAE group post-exercise (Supplementary Figure S3F, F = 4.263, p = 0.0205, η2 (p) = 0.165). Pearson’s correlation analysis revealed a significant positive correlation between the ratio of post-to pre-exercise reaction time in the word test of the Stroop task and the HbR concentration in channel 45 post-exercise (Supplementary Figure S3I, Pearson r = 0.3843, *p* = 0.0084), an association that was absent pre-exercise (Supplementary Figure S3J, Pearson r = −0.0932, *p* = 0.05417). These results suggest that low-intensity aerobic exercise enhances cortical activation in regions such as S1 and the left Broca’s area during the Stroop word test. Moreover, the decreased HbR concentration in channel 45 post-exercise correlates with the exercise-induced improvement in Stroop word-test reaction time, an association absent before exercise, indicating its emergence as a potential consequence rather than a predictive marker of the effect.

Lastly, no significant change of HbR concentration was observed in the color test of the Stroop task among the three groups pre-exercise (Supplementary Figure S2I–L) and post-exercise (Supplementary Figure S3K–M). However, a diminishing trend was apparent in the LAE group as compared with the SE and MAE groups post-exercise (Supplementary Figure S3K–M), which suggests that as in the Stroop word test, low-intensity exercise enhanced cortical activation during the Stroop color test.

### Cardiopulmonary function

**FIGURE 5:**
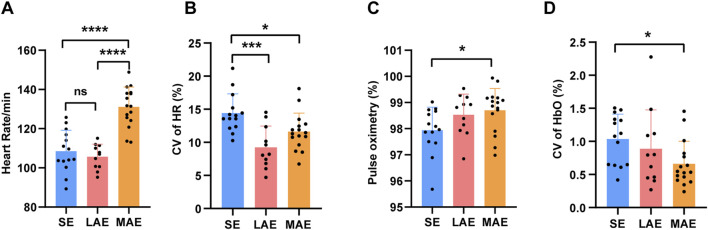
Cardio-pulmonary function during exercise. **(A–D)** The averaged percentage of heart rate **(A)**, the coefficient of variation of heart rate **(B)**, the pulse oximetry **(C)**, the coefficient of variation of pulse oximetry **(D)** of three groups during 10-min exercise. n = 14 for SE group, 11 for LAE group and 16 for MAE group, one-way ANOVA. HR: heart rate; CV: coefficient of variation. *p < 0.05, **p < 0.01, ***p < 0.001, ****p < 0.000, error bars indicate SD.

Several established physical exercise studies have emphasized heart rate and its variability as indicators of exercise intensity and VO_2_max (Achten and Jeukendrup, 2003). To investigate whether cardiopulmonary function serves as a predictor of the cognitive benefits derived from exercise, we monitored heart rate and blood oxygen: The HR per minute during a 10-min exercise session were significantly higher in the MAE group than in both the LAE and SE groups, with no discernible difference between the LAE and SE groups (Figure 5A, F = 31.05, p < 0.0001, η2 (p) = 0.620), whereas the coefficient of variation (CV) in HR was greater in the SE group than in the LAE and MAE groups (Figure 5B, F = 9.55, p = 0.0004, η2 (p) = 0.334). Moreover, pulse oximetry revealed a slight but significant increase in blood oxygen concentration, accompanied by a reduction in its CV, in the MAE group (Figures 5C,D). However, subsequent Pearson’s correlation analysis failed to uncover any statistically significant correlation between the ratio of post-to pre-exercise reaction time in the Stroop task and the HR or its CV (HR: Pearson r = 0.02990, *p* = 0.8528, CV of HR: Pearson r = 0.2679, *p* = 0.0904). These results did not provide definitive evidence for a direct relationship between HR dynamics and the cognitive improvement associated with exercise, suggesting that other factors potentially contribute to the observed cognitive enhancement. Furthermore, because of equipment malfunction during the experiment, we encountered a loss of real-time heart rate and blood oxygen data in the case of certain participants, which resulted in a discrepancy in sample sizes across groups as compared to the samples sizes used above.

### Gait and pose analysis

**FIGURE 6:**
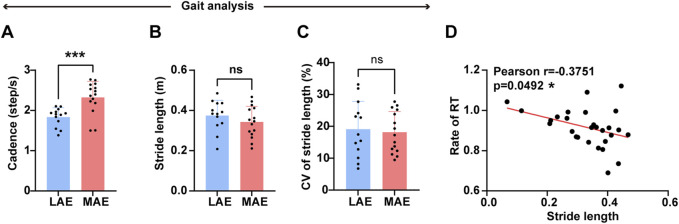
Gait and pose analysis between low-tensity and moderate-tensity aerobic exercise groups. **(A–C)** The cadence **(A)**, normalized stride length **(B)**, and coefficient of variation of stride length **(C)** between LAE and MAE groups during 10-min exercise. n = 13 for LAE group and 15 for MAE group, unpaired *t*-test. CV: coefficient of variation. **(D)** The correlation analysis between the ratio of post-to pre-exercise reaction time in the Stroop task and the normalized stride length of two aerobic exercise groups during exercise. n = 13 for LAE group and 15 for MAE group, Pearson’s correlation. *p < 0.05, **p < 0.01, ***p < 0.001, ****p < 0.000, error bars indicate SD.

We next sought to predict the cognitive benefits of aerobic exercise by integrating gait and pose analysis. Before the data analysis, we excluded samples that lacked cognitive data or showed poor video quality. As anticipated, we observed a significant increase in cadence between LAE and MAE groups (Figure 6A, t = 3.887, p = 0.0006, η2p = 0.296). Notably, stride length and its CV did not differ significantly between the LAE and MAE groups (Figures 6B,C, stride length: t = 1.107, p = 0.2784, η2p = 0.076, CV of stride length: t = 0.3287, p = 0.745, η2p = 761), although stride length showed a slight increasing trend in the LAE group relative to the MAE group (Figure 6B). Intriguingly, a significant negative correlation was found between the ratio of post-to pre-exercise reaction time in the Stroop task and the stride length in the two groups (Figure 6D, Pearson r = −0.3751, *p* = 0.0492), suggesting that a greater stride length is potentially indicative of improved cognitive benefits.

### Cortical functional connection during exercise

**FIGURE 7:**
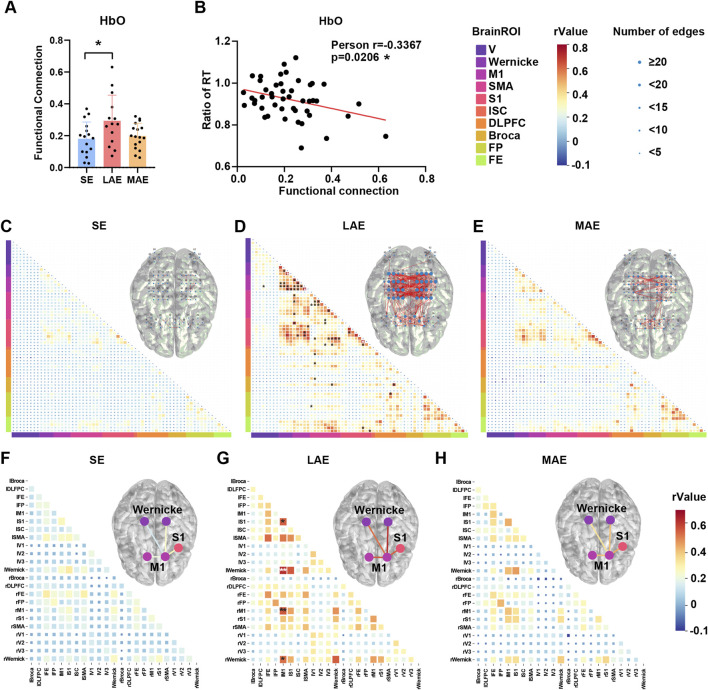
Effects of different exercises on fNIRS functional connection of HbO. **(A)** The averaged functional connectivity coefficient of HbO among three groups during 10-min exercise. n = 16 for SE group, 14 for LAE group and 17 for MAE group, One-way ANOVA. **(B)** The relationship between the ratio of post-to pre-exercise reaction time in the Stroop task and the averaged functional connection of HbO of three group during exercise. n = 16 for SE group, 14 for LAE group and 17 for MAE group, Pearson’s correlation. *p < 0.05, **p < 0.01, ***p < 0.001, ****p < 0.000; error bars indicate SD. **(C–E)** Channel-wise functional connection of HbO for SE group **(C)**, LAE group **(D)** and MAE group **(E)** during 10-min exercise. The bottom triangle represents the r value of channel-wise functional connection, and the upper triangle represents the brain channel network connection. The size of a node indicates how many edges which r value >0.4 are connected to this channel. n = 16 for SE group, 14 for LAE group and 17 for MAE group, Pearson’s correlation with FDR correction applied. **(F–H)** Region-wise functional connection of HbO for SE group **(F)**, LAE group **(G)** and MAE group **(H)** during 10-min exercise. The bottom triangle represents the r value of region-wise functional connection, and the upper triangle represents the brain region network connection. The color of line represents the r value of region-wise functional connection. n = 16 for SE group, 14 for LAE group and 17 for MAE group, Pearson’s correlation with FDR correction applied. * FDR-corrected p < 0.05, ** FDR-corrected p < 0.01.

In contrast to other neuroimaging modalities, fNIRS presents a unique advantage in assessing cortical activation during exercise due to its minimal susceptibility to artifacts. By using a 63-channel fNIRS system, we obtained results showing a significant elevation in the mean functional connectivity coefficient of cortical HbO during low-intensity aerobic exercise as compared with stretching exercise, an effect that was not observed with moderate-intensity aerobic exercise (Figure 7A, F = 3.962, p = 0.0262, η2 (p) = 0.152). Importantly, the mean functional connectivity coefficient of cortical HbO negatively correlated with the change in Stroop task reaction time post-exercise (Figure 7B, Pearson r = −0.3367, *p* = 0.0206), suggesting that a more strongly activated cortical functional network could predict cognitive benefits associated with exercise. Additionally, there was no significant correlation between functional connectivity and HR (neither baseline [Pearson r = 0.06013, p = 0.7124] or during exercise [Pearson r = −0.007015, p = 0.9657]) or Stride length (Pearson r = 0.2048, p = 0.3865), which, to a certain extent, rules out the influence of cardiovascular effects. When comparing functional connectivity patterns across groups, the LAE group exhibited a greater number of channel-to-channel connections, particularly in the parietal cortex, as compared with both the SE and MAE groups (Figure 7D, upper). These parietal cortex connections encompassed regions such as Wernicke’s area, primary motor cortex (M1), pre-motor and supplementary motor cortex (SMA), primary somatosensory cortex (S1), left subcentral area (lSC), parts of Broca’s area, and frontal eye field (FE) (Figure 1C), with notable connectivity of certain channels between M1 and Wernicke’s area, SMA, and S1, between SMA and S1, between S1 and FE, and between Broca’s area and SMA and DLPFC in the LAE group relative to the SE group (Figures 7C,D, bottom). Furthermore, region-wise functional connectivity analysis of HbO revealed significantly enhanced interregional connectivity within the network, particularly between left M1 and right M1, left and right Wernicke’s area, and left S1 (Figure 7G). However, no significant differences were detected between the MAE and SE groups following FDR correction (Figures 7C,E,F, and H).

**FIGURE 8:**
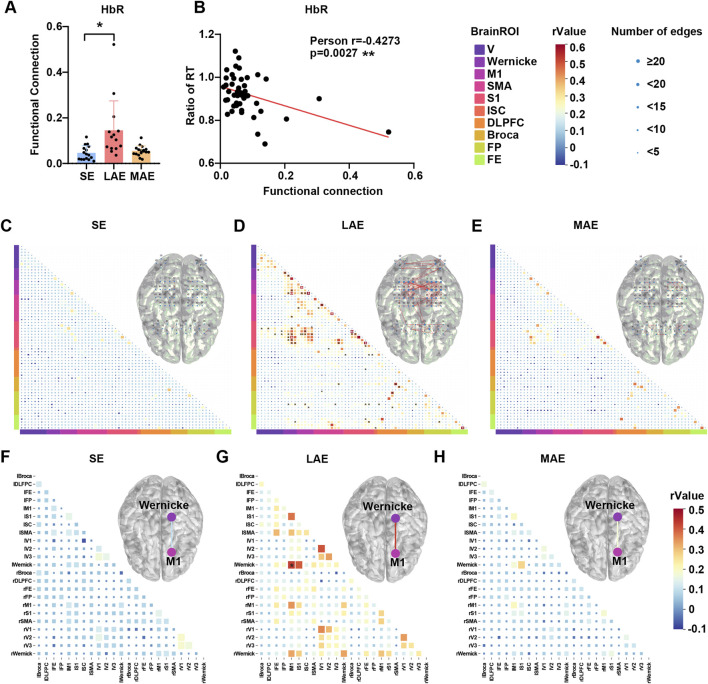
Effects of different exercises on fNIRS functional connection of HbR. **(A)** The averaged functional connection of HbR among three groups during 10-min exercise. n = 16 for SE group, 14 for LAE group and 17 for MAE group, One-way ANOVA. **(B)** The relationship between the ratio of post-to pre-exercise reaction time in the Stroop task and the averaged functional connection of HbR of three group during 10-min exercise. n = 16 for SE group, 14 for LAE group and 17 for MAE group, Pearson’s correlation. *p < 0.05, **p < 0.01, ***p < 0.001, ****p < 0.000; error bars indicate SD. **(C–E)** Channel-wise functional connection of HbR for SE group **(C)**, LAE group **(D)** and MAE group **(E)** during 10-min exercise. The bottom triangle represents the r value of channel-wise functional connection, and the upper triangle represents the brain network connection. The size of a node indicates how many edges which r value >0.3 are connected to this channel. n = 16 for SE group, 14 for LAE group and 17 for MAE group, Pearson’s correlation with FDR correction applied. **(F–H)** Region-wise functional connection of HbR for SE group **(F)**, LAE group **(G)** and MAE group **(H)** during 10-min exercise. The bottom triangle represents the r value of region-wise functional connection, and the upper triangle represents the brain region network connection. The color of line represents the r value of region-wise functional connection. n = 16 for SE group, 14 for LAE group and 17 for MAE group, Pearson’s correlation with FDR correction applied. * FDR-corrected p < 0.05, ** FDR-corrected p < 0.01.

Similarly, examination of the mean functional connectivity coefficient of cortical HbR revealed a notable augmentation of cortical functional connectivity during low-intensity aerobic exercise (Figure 8A, F = 3.853, p = 0.0356, η2 (p) = 0.268), accompanied by a statistically significant positive association between the cognitive gains derived from exercise and the functional connectivity coefficient (Figure 8B, Pearson r = −0.4273, *p* = 0.0027). And no significant correlation between functional connectivity and HR (neither baseline [Pearson r = −0.004487, p = 0.9781] or during exercise [Pearson r = −0.2441, p = 0.1290]) or Stride length (Pearson r = 0.2527, p = 0.2824) was found, which exclude a cardiovascular effect. In-depth analysis of channel-wise functional connectivity of HbR uncovered a marked elevation in functional coupling between M1 and Wernicke’s area and M1 itself, and between S1 and the visual cortex (V), Wernicke’s area, M1, SMA, and S1 in the LAE group (Figure 8D, bottom). Moreover, channel-to-channel connectivity was augmented within the dorsal parietal cortex and the right-sided frontal and occipital cortices (Figure 8D, upper). Region-wise functional connectivity analysis of HbR further underscored a significant enhancement in connectivity between left M1 and left Wernicke’s area in the LAE group (Figure 8G). By contrast, no statistically significant differences emerged between the MAE and SE groups after FDR correction (Figures 8C,E,F and H). In conclusion, these results highlight the potential of fNIRS in elucidating the neural mechanisms underlying exercise-induced cognitive improvements.

## Discussion

Here, we observed a notable augmentation in Stroop reaction time, a key indicator of executive function, subsequent to low-intensity aerobic exercise as compared to moderate-intensity exercise and stretching exercise. This enhancement was accompanied by greater stride length and heightened functional connectivity within the parietal cortex, particularly between the left M1 and nearby brain regions such as left S1, right M1, and Wernicke’s area. Our findings support the hypothesis that during low-intensity aerobic exercise, the upregulation of neural activity involved in stride modulation, which is intricately linked with motor learning and M1 plasticity-mediated cortical network dynamics, strengthens parietal functions and further fosters underlying cognitive enhancement.

Our study revealed that low-intensity aerobic exercise demonstrated faster reaction times in the Stroop task compared to stretching exercises and moderate-intensity aerobic exercise. This finding raises two fundamental questions regarding the mechanisms underlying LAE’s cognitive superiority and its selective impact on reaction time rather than other cognitive measures. (1) The cognitive advantage of LAE appears to derive from three integrated neurophysiological mechanisms. First, LAE selectively enhances functional connectivity within parieto-motor networks (left M1-left S1, right M1 and Wernicke’s area), optimizing sensorimotor integration while minimizing prefrontal resource competition (Dietrich, 2006). Second, by maintaining exertion below the lactate threshold (RPE [Rating of Perceived Exertion] 10-13 vs. 14-18 for moderate exercise) (Scherr et al., 2013; Ji et al., 2022), LAE preserves metabolic homeostasis, preventing adenosine-mediated central fatigue (Nybo and Secher, 2004) while sustaining cerebral oxygenation (Ochi et al., 2022). Third, the dissociative self-talk during LAE promotes attentional resource conservation for subsequent executive processing (St Clair Gibson and Foster, 2007). (2) The selective improvement in Stroop reaction time, rather than task accuracy or performance on verbal fluency/n-back tests, reflects both task-specific and population-specific factors. The Stroop task’s reliance on prefrontal (DLPFC, middle frontal gyrus and frontopolar area) inhibitory control circuits makes it particularly sensitive to exercise-induced modulation of BDNF signaling and oxygenation (Ji et al., 2023; Liu et al., 2023; Kvist et al., 2024; Wu et al., 2024). Furthermore, young adults’ intact neural reserves create ceiling effects for accuracy measures while preserving reaction time sensitivity to acute exercise interventions. In contrast, verbal fluency and working memory tasks engage distinct neural networks less responsive to transient physiological changes (Wurz et al., 2021; Zheng et al., 2022). Crucially, stretching controls confirmed effect specificity: Identical task exposure yielded no Stroop improvement (Figures 3A–C), excluding practice effects despite comparable heart rates (Figure 5A). This underscores that rhythmic coordination in aerobic exercise (vs. isolated stretching) drives cognitive benefits through integrated sensorimotor pathways.

Several studies have documented a robust correlation between deteriorated gait characteristics, encompassing reduced walking speed, diminished stride length, elevated stride length variability, and lower cadence, with declining cognitive function in the elderly population, particularly among people with mild cognitive impairment (MCI), Alzheimer’s disease (Allali et al., 2016; Kuan et al., 2021; He et al., 2024; Nester et al., 2024), Parkinson’s disease (Stegemoller et al., 2014; Monaghan et al., 2023), and Wilson’s disease (Wang et al., 2023), and have further suggested these gait impairments as potential predictive markers for dementia onset (Beauchet et al., 2016). Importantly, interventions aimed at enhancing gait function, such as treadmill walking programs, moderate physical activity regimens, resistance exercise training, and dual-modality cognitive-gait training protocols, have shown the capacity to improve both gait performance and cognitive abilities, notably in domains of processing speed and executive function (mediated by DLPFC, anterior cingulate cortex and frontoparietal network) (Fritz et al., 2015; Yoon et al., 2018; Chai et al., 2023; Galle et al., 2023; Sasikumar et al., 2023). These findings collectively underscore the intricate interplay between cognitive decline and the affected brain regions, suggesting a shared neural mechanism at the core of gait abnormalities. Despite these advances, a critical knowledge gap persists regarding the mechanisms underlying the enhancement of cognitive function through intensified exercise training and improved gait performance. Addressing this gap necessitates rigorous scientific inquiry to unravel the complex interplay among physical activity, gait optimization, and cognitive faculties. Here, we comprehensively analyzed various gait parameters, encompassing cadence, stride length and its variability, stride width, with the primary objective of identifying the most sensitive and predictive correlation of exercise-induced cognitive enhancement. Intriguingly, our results showed that stride length is the paramount predictor of cognitive benefits when directly comparing low-intensity with moderate-intensity aerobic exercise. Critically, while our randomized intervention design (exercise → gait/neural changes → cognition) provides temporally plausible evidence for a potential causal pathway linking stride length to cognitive enhancement, we acknowledge two key limitations in causal inference: First, stride length was not experimentally isolated from other exercise-induced physiological effects; Second, although both stride length increases and parietal connectivity enhancements independently correlated with Stroop improvement, we observed no significant direct correlation between these two mediators. This suggests that stride length and parietal connectivity may operate through parallel, complementary pathways rather than a linear mediational chain (e.g., stride length → parietal connectivity → cognition). To definitively establish causality, future studies should employ: (1) Stride-Length-Targeted Interventions using real-time gait biofeedback to selectively modulate stride length while controlling speed/cadence; (2) Dual-Task Paradigms probing cognitive load during stride manipulation to isolate neural resource allocation; and (3) Causal Network Analysis integrating multimodal neuroimaging (e.g., fMRI effective connectivity) with longitudinal gait kinematics to dissect directional influences among motor efficiency, neural plasticity, and cognitive outcomes. Notably, this significant difference was not observed in the analysis of walking speed and cadence, underscoring the unique role of stride length in mediating the relationship between exercise intensity and cognitive enhancement. This observation stands in contrast to the findings of previous studies, highlighting the need for further investigation into the nuanced relationships between gait characteristics and cognitive outcomes.

Previously, the cognitive enhancements elicited by exercise, whether acute or chronic, were predominantly investigated through comparative analyses of pre- and post-intervention changes in cognitive performance metrics, exemplified by the Stroop task, in conjunction with neuroimaging techniques, which were frequently affected by technological constraints. These studies consistently identified activation patterns within select brain regions, notably the DLPFC, left orbitofrontal cortex, left Broca’s area, left S1, anterior cingulate cortex, and prefrontal cortex (Herold et al., 2018; Thomas et al., 2020; Hu et al., 2021; Yang et al., 2022; You et al., 2023), locating in the prefrontal cortex and hippocampus, which have been be functionally implicated in emotional regulation and cognitive processes such as attention, information processing, and memory. Recently, the use of fNIRS in dual-task paradigms, where cycling or walking was concurrently performed with cognitive assessments, has yielded intriguing insights. In younger adults, activation of the contralateral motor cortex was augmented during dual-task engagement (Tempest and Reiss, 2019), whereas in older adults, contrasting patterns were observed, including reduced activation in the frontopolar area, DLPFC, right SMA (Zheng et al., 2022), and prefrontal cortex (Baek et al., 2023). Moreover, in people with MCI, diminished activation was reported in the premotor, motor, and prefrontal cortex (Talamonti et al., 2022), highlighting the potential sensitivity of these brain areas to exercise-induced modulation and their role in cognitive improvement. However, the dual-task experimental design introduces confounding factors, making it unclear whether the activation of these brain regions stems from the cognitive process, motor process, or an interactive effect between the two. Consequently, ascertaining which brain regions’ activities mediate exercise-induced cognitive enhancement becomes challenging, further complicating the determination of causality. In this study, as in other research endeavors, we devised an fNIRS experimental protocol to compare cognitive function before and after exercise, and we also incorporated the monitoring of cortical activity during distinct motor processes to enrich our understanding. Our results revealed a pronounced enhancement in the activation of S1, left Broca’s area, and right DLPFC during the Stroop task post-exercise, mirroring findings from other studies. However, these indices failed to exhibit a statistically significant correlation with cognitive improvement, which suggests the involvement of alternative underlying mechanisms in exercise-mediated cognitive benefits. Conversely, the overall functional connectivity of HbO and HbR during exercise displayed a marked positive association with accelerated execution speed. Notably, relative to stretching exercise, low-intensity aerobic exercise, which yielded the most substantial improvement in Stroop reaction time, induced a significant enhancement in functional connectivity within the parietal cortex, encompassing M1, SMA, S1, and Wernicke’s area. In ROI connectivity analysis, we observed heightened connectivity between the left M1 and right M1, left and right Wernicke’s area, and left S1 induced by low-intensity aerobic exercise. These brain regions are intimately linked to the process of contralateral voluntary movement, contralateral sensation, language generation, memory, and fluency. A recent meta-analysis revealed that physical exercise interventions elicit distinct alterations in functional activation patterns, primarily centered within the precuneus region and intimately tied to the frontoparietal, dorsal attention, and default mode networks (Yu Q. et al., 2021). Intriguingly, persons undergoing exercise training, even people with Parkinson’s disease, show augmented activation of the left M1 and SMA, alongside enhanced functional connectivity of the right posterior cingulate gyrus and right central operculum (Green et al., 2018). This increased activation of left M1 and left SMA and further functional connectivity of the right posterior cingulate gyrus and right central operculum have also been recorded in individuals with exercise training and Parkinson’s disease (Messa et al., 2019). However, our study design has limitations that warrant consideration. Although we implemented an 8-min rest period before post-exercise cognitive tests to standardize physiological recovery (with heart rate normalization confirmed) (Herold et al., 2018), it remains unclear whether this fixed interval equally accommodates inter-individual variability in cognitive fatigue dissipation or cerebrovascular recovery kinetics. While our heart rate-guided exercise intensity and post-exercise monitoring mitigated systemic confounds, subtle differences in cardiorespiratory fitness or biological factors may still influence neural readiness across participants. These findings suggest a potential mechanism whereby acute physical exercise fosters cognitive enhancement by stimulating M1 plasticity, a pivotal process underpinning motor learning. It should be noted that as a single-session intervention study, our findings are limited to acute neurophysiological responses and cannot directly infer long-term cognitive benefits or structural neuroplastic changes, which typically require sustained exercise regimens (e.g., ≥8-week interventions). Furthermore, this enhancement might be mediated through modulation of gamma-aminobutyric acid-mediated inhibition (Cirillo, 2021), facilitating neural communication within proximal brain regions such as the SMA, S1, and Wernicke’s area. Critically, the concordant enhancement of functional connectivity between left M1 and Wernicke’s area—observed in both HbO and HbR signals during low-intensity aerobic exercise—provides robust hemodynamic validation of this neural coupling. This dual-indicator agreement confirms biological specificity by linking neurovascular coupling (HbO) with metabolic demand (HbR), rules out motion artifacts as a confounder, and establishes exercise-phase specificity (distinct from post-exercise Stroop effects). Neurofunctionally, we reinterpret this connectivity through the lens of embodied self-regulation. During low-intensity aerobic exercise—characterized by dissociative, non-instructive self-talk (St Clair Gibson and Foster, 2007)—heightened M1-Wernicke’s co-activation may liberate cognitive resources from explicit motor monitoring, prime procedural memory networks via spontaneous action rehearsal (e.g., subconscious pedaling rhythm optimization) and accelerate sensorimotor predictions through temporal gap utilization (“temporal wedge”). This repositions Wernicke’s area as a supermodel integrative node for movement automatization, independent of language task demands. Ultimately, the complex neuronal network underlying these exercise-induced changes extends to regions associated with cognition, including the prefrontal cortex and hippocampus, thereby suggesting a multifaceted pathway through which physical exercise could positively impact cognitive function.

In the initial phase of our study, we also hypothesized that the cognitive enhancement induced by exercise would positively correlate with indices such as exercise intensity, walking speed, heart rate and its variability during exercise, and cortical brain activity levels. However, contrary to our expectations, we found that a single session of low-intensity aerobic exercise outperformed moderate-intensity exercise in improving executive functions in healthy young adults. It should be noted that the lack of objective cardiorespiratory fitness indicators (e.g., VO_2_max, blood lactate) may limit the precision in defining exercise intensity and reduce generalizability to athletic or clinical populations. Moreover, this exercise-induced cognitive enhancement was associated with increased stride length and enhanced functional connectivity of motor and sensory brain regions within the parietal cortex. Although physical activity guidelines widely recommend moderate-intensity aerobic exercise for cognitive enhancement (Petersen et al., 2018; Bull et al., 2020), the dose-response relationship in exercise-induced cognitive improvement remains debated (Hoffmann et al., 2016; Chu et al., 2017; Stern et al., 2019; Law et al., 2020; Yu F. et al., 2021; Gallardo-Gomez et al., 2022; Tarumi et al., 2022). Numerous studies in this field, comparing various intensities, types, and total volumes of exercise, have yielded conflicting conclusions, potentially suggesting that these exercise parameters are not crucial predictors of cognitive improvement. Our findings suggest the intriguing conclusion that exercises that elicit greater stride length and exert a more pronounced effect on the activation of specific parietal cortical regions, such as M1, S1, SMA, and Wernicke’s area, might be more effective than other forms of exercise in enhancing cognitive functions across diverse populations.

Lastly, certain points regarding the generalization of our findings should be considered. First, despite our meticulous efforts to enroll a cohort of healthy young adults and to achieve baseline equilibration across the three exercise groups in terms of sex, age, cognitive function, and physical fitness, the generalizability of our study population remains constrained. Notably, we also did not examine the effect of high-intensity aerobic exercise (defined as THR exceeding 70% of HRmax), and this limits the scope of the insights obtained in the study. However, based on existing evidence, we hypothesize an inverted-U relationship between exercise intensity and cognitive benefits, where excessive intensity (>70% HRmax) may impair cognition through neural resource depletion and sensorimotor-cognitive interference pathways (Dietrich, 2006; You et al., 2023; Yuan et al., 2025). Importantly, our findings may hold particular relevance for aging and neurodegenerative populations. The low-intensity aerobic exercise (<50% HRmax) that demonstrated cognitive benefits in our study offers critical clinical advantages: its reduced joint loading minimizes fall risk while improving gait parameters (e.g., stride length enhancement), a finding particularly relevant for addressing motor symptoms in Parkinson’s disease or Alzheimer’s disease. The parietal-motor connectivity (M1-SMA, S1, Wernicke’s area network) enhanced by low-intensity exercise demonstrates relative preservation during aging compared to vulnerable prefrontal networks (Heuninckx et al., 2005; Seidler et al., 2010; Michely et al., 2018), suggesting its potential as a compensatory target for cognitive-motor interventions in older adults. Consequently, future studies should strive to broaden the applicability of our findings by including middle-aged and elderly adults, as well as populations with diverse comorbidities; Examining a wider range of exercise intensities to validate the hypothesized inverted-U dose-response curve.

Second, the inherent technical constraints of fNIRS limited our investigation to cortical hemodynamics, precluding direct assessment of deep brain structures, e.g., hippocampus and posterior cortical regions such as the precuneus and temporal lobes. This omission restricts characterization of hippocampal contributions to memory consolidation and precuneus-mediated attentional integration, thereby narrowing the interpretative scope of exercise-induced modulations in these networks. Additionally, while we employed Pearson’s correlation for functional connectivity analysis with FDR correction and effect size thresholds, the limited spatial resolution of fNIRS raises questions about the precise localization of signals—particularly in adjacent cortical areas like M1 and S1 where partial volume effects may blur functional boundaries. Our anatomical co-registration and >50% channel contribution criteria mitigate this concern, but residual uncertainty persists. While our focus on parietal-motor connectivity aligns with fNIRS’s motion-compatibility advantages, future multimodal studies integrating fMRI are warranted to resolve subcortical and deep cortical dynamics.

Third, while our intervention design identifies stride length rather than cadence or walking speed as a specific correlate of exercise-induced cognitive improvement, we cannot experimentally dissociate stride length’s independent causal role from its inherent coupling with neuromuscular coordination mechanisms. Although our functional connectivity data implicate parietal-motor networks and suggest a threshold effect of cortico-cerebellar efficiency, these findings remain indirect evidence for neuro-metabolic mechanisms, lacking direct validation through neurochemical or electrophysiological measures. Furthermore, the observed cognitive-motor coupling may exhibit task specificity, as our conclusions derive primarily from Stroop test performance; whether this relationship generalizes to other cognitive domains requires systematic investigation. Future studies should employ real-time gait biofeedback to isolate stride length effects and integrate multimodal neuroimaging to resolve metabolic underpinnings of these behavioral associations.

## Data Availability

The raw data supporting the conclusions of this article will be made available by the authors, without undue reservation.
