# Supplementary material for: Acute effect of low-intensity aerobic exercise on eliciting enhanced parietal activation and promoting executive function performance more than moderate-intensity exercise
Source: Front Physiol. 2025 Aug 18;16:1581481. doi: 10.3389/fphys.2025.1581481 (PMC12399649; doi:10.3389/fphys.2025.1581481)
Supplement: Supplementary file 1 [file DataSheet1.pdf]

Supplemental table and figure legends

Table S1 The MNI coordinates and Brodmann area (Chris rorden' MRIcro) of the 63-channels fNIRS

fNIRS 63-channels			MNI coordinates			ROI	The Brodmann area (Chris rorden' MRIcro)
Channel	Source	Detector	X	Y	Z		
CH1	S1	D1	52.715	-49.594	57.027	rWernick	40 - Supramarginal gyrus part of Wernicke's area
CH2	S1	D12	40.479	-48.102	67.799	rWernick	40 - Supramarginal gyrus part of Wernicke's area
CH3	S2	D2	52.223	46.059	-6.227	rDLPFC	46 - Dorsolateral prefrontal cortex
CH4	S2	D7	58.608	33.374	5.6619	rBroca	45 - pars triangularis Broca's area
CH5	S3	D2	40.048	63.163	-2.7213	rFP	10 - Frontopolar area
CH6	S3	D3	17.549	72.758	-0.1046	rFP	10 - Frontopolar area
CH7	S3	D8	29.818	66.704	12.564	rFP	10 - Frontopolar area
CH8	S4	D3	-13.37	73.25	-0.4599	lFP	10 - Frontopolar area
CH9	S4	D4	-39.423	63.46	-2.3843	lFP	10 - Frontopolar area
CH10	S4	D9	-27.188	67.607	14.253	lFP	10 - Frontopolar area
CH11	S5	D4	-53.249	43.869	-8.8088	lDLPFC	46 - Dorsolateral prefrontal cortex
CH12	S5	D10	-59.017	29.449	1.3004	lBroca	45 - pars triangularis Broca's area
CH13	S6	D5	-58.059	-49.415	52.961	lWernick	40 - Supramarginal gyrus part of Wernicke's area
CH14	S6	D21	-	-	52.8	lS1	3 - Primary Somatosensory Cortex

			58.879	25.635	69		
CH15	S6	D22	-67.792	-29.713	41.573	lS1	2 - Primary Somatosensory Cortex
CH16	S7	D12	30.634	-46.967	74.004	rS1	1 - Primary Somatosensory Cortex
CH17	S7	D19	20.471	-44.015	79.599	rS1	1 - Primary Somatosensory Cortex
CH18	S8	D2	46.96	53.356	9.4949	rDLPFC	46 - Dorsolateral prefrontal cortex
CH19	S8	D7	52.444	39.396	22.172	rBroca	45 - pars triangularis Broca's area
CH20	S8	D8	38.609	55.042	24.157	rDLPFC	46 - Dorsolateral prefrontal cortex
CH21	S9	D3	2.7632	68.674	15.392	FP	10 - Frontopolar area
CH22	S9	D8	15.885	64.488	27.918	rFP	10 - Frontopolar area
CH23	S9	D9	-13.63	64.139	30.296	lFP	10 - Frontopolar area
CH24	S10	D4	-48.787	50.915	7.0349	lDLPFC	46 - Dorsolateral prefrontal cortex
CH25	S10	D9	-37.949	54.728	25.726	lDLPFC	46 - Dorsolateral prefrontal cortex
CH26	S10	D10	-54.232	36.282	19.055	lBroca	45 - pars triangularis Broca's area
CH27	S11	D21	-57.85	-5.737	50.637	lSMA	6 - Pre-Motor and Supplementary Motor Cortex
CH28	S11	D22	-64.606	-5.9163	37.809	lSC	43 - Subcentral area
CH29	S11	D23	-51.412	19.724	43.65	lBroca	44 - pars opercularis_ part of Broca's area
CH30	S12	D12	34.414	-23.983	72.973	rM1	4 - Primary Motor Cortex
CH31	S12	D14	21.8	-	75.9	rSMA	6 - Pre-Motor and Supplementary

				2.4343	7		Motor Cortex
CH32	S12	D19	22.472	-23.871	77.03	rM1	4 - Primary Motor Cortex
CH33	S12	D20	33.636	-4.1696	66.606	rSMA	6 - Pre-Motor and Supplementary Motor Cortex
CH34	S13	D17	49.963	20.335	45.549	rDLPFC	9 - Dorsolateral prefrontal cortex
CH35	S13	D20	42.718	19.255	54.791	rDLPFC	9 - Dorsolateral prefrontal cortex
CH36	S14	D15	-36.705	-2.9302	65.42	lSMA	6 - Pre-Motor and Supplementary Motor Cortex
CH37	S14	D18	-34.897	18.097	61.338	lFE	8 - Includes Frontal eye fields
CH38	S14	D21	-47.349	-3.0424	58.501	lSMA	6 - Pre-Motor and Supplementary Motor Cortex
CH39	S14	D23	-43.036	18.69	54.752	lDLPFC	9 - Dorsolateral prefrontal cortex
CH40	S15	D15	-25.896	-3.5356	74.138	lSMA	6 - Pre-Motor and Supplementary Motor Cortex
CH41	S15	D18	-21.592	18.607	67.686	lFE	8 - Includes Frontal eye fields
CH42	S16	D6	49.155	-86.432	3.8738	rV3	19 - V3
CH43	S16	D13	37.677	-92.245	20.031	rV3	19 - V3
CH44	S17	D1	55.873	-25.241	57.015	rS1	1 - Primary Somatosensory Cortex
CH45	S17	D12	46.155	-23.948	66.934	rM1	4 - Primary Motor Cortex
CH46	S17	D17	55.923	-4.0537	52.341	rSMA	6 - Pre-Motor and Supplementary Motor Cortex
CH47	S17	D20	44.6	-	61.2	rSMA	6 - Pre-Motor and Supplementary

			68	3.0585	33		Motor Cortex
CH48	S18	D1	66.323	-26.836	45.282	rS1	1 - Primary Somatosensory Cortex
CH49	S18	D17	62.894	-3.5529	39.18	rSMA	6 - Pre-Motor and Supplementary Motor Cortex
CH50	S19	D14	21.828	18.253	67.749	rFE	8 - Includes Frontal eye fields
CH51	S19	D20	32.577	17.929	61.571	rFE	8 - Includes Frontal eye fields
CH52	S20	D5	-46.195	-47.237	61.975	lWernick	40 - Supramarginal gyrus part of Wernicke's area
CH53	S20	D11	-35.683	-45.36	71.568	lS1	1 - Primary Somatosensory Cortex
CH54	S20	D15	-38.361	-24.443	70.516	lM1	4 - Primary Motor Cortex
CH55	S20	D21	-50.39	-25.629	65.032	lS1	3 - Primary Somatosensory Cortex
CH56	S21	D11	-23.582	-44.571	76.207	lS1	1 - Primary Somatosensory Cortex
CH57	S21	D15	-27.595	-22.541	76.402	lM1	4 - Primary Motor Cortex
CH58	S22	D6	36.101	-97.206	-11.504	rV2	18 - Visual Association Cortex (V2)
CH59	S22	D13	24.717	-104.84	5.6226	rV1	17 - Primary Visual Cortex (V1)
CH60	S23	D16	-26.694	-104.11	2.7701	lV1	17 - Primary Visual Cortex (V1)
CH61	S23	D24	-38.432	-92.72	16.426	lV3	19 - V3
CH62	S24	D16	-34.89	-97.831	-12.207	lV2	18 - Visual Association Cortex (V2)
CH63	S24	D24	-	-	-	lV3	19 - V3

47.1<br>47	89.0<br>9	0.03<br>08

Table S2 Physical history of all participants

		Control exercise	Aerobic exercise		
		Stretching exercise (n=17)	Low-intensity (n=15)	Moderate-intensity (n=17)	*p* value
Exercise duration (min/week)	Low (<150)	9/52.9%	10/66.7%	9/52.9%	0.670
	High (>150)	8/47.1%	5/33.3%	8/47.1%	
Daily step count	Low (<10000)	13/76.5%	9/60.0%	9/52.9%	0.346
	Low (>10000)	4/23.5%	6/40.0%	8/47.1%	

Table S3 Negative results of cognitive response

Trail	Factor	Effect	Df	F (DFn, DFd)	P value	Mean pre-exercise	Mean post-exercise
Stroop task	Accuracy of total	Interaction	2	F (2, 44) = 2.482	P=0.0952	SE = 0.9631 LAE = 0.9521 MAE = 0.9735	SE = 0.9706 LAE = 0.9614 MAE = 0.9571
		Group	2	F (2, 44) = 0.6259	P=0.5395		
		Time	1	F (1, 44) = 0.0003817	P=0.9845		
	Accuracy of word test	Interaction	2	F (2, 44) = 4.452	P=0.0173	SE = 0.9531 LAE = 0.9400 MAE = 0.9841	SE = 0.9725 LAE = 0.9693 MAE = 0.9688
		Group	2	F (2, 44) = 2.708	P=0.0778		
		Time	1	F (1, 44) = 2.941	P=0.0934		
	Accuracy of color test	Interaction	2	F (2, 44) = 0.07737	P=0.9257	SE = =0.9744 LAE = 0.9636 MAE = 0.9594	SE = 0.9663 LAE = =0.9486 MAE = 0.9435
		Group	2	F (2, 44) = 1.244	P=0.2981		
		Time	1	F (1, 44) = 2.115	P=0.1529		
Ratio of Incon/con in Stroop task	Accuracy of word test	Interaction	2	F (2, 44) = 0.8275	P=0.4438	SE = 0.9920 LAE =	SE = 0.9778 LAE =
		Group	2	F (2, 44) =	P=0.0025		

				6.908		0.9571 MAE = 0.9853	0.9250 MAE = 0.9853
		Time	1	F (1, 44) = 2.323	P=0.1347		
	Reaction time of word test	Interaction	2	F (2, 44) = 1.872	P=0.1659	SE = 1.096 LAE = 1.046 MAE = 1.045	SE = 1.121 LAE = 1.077 MAE = 1.000
		Group	2	F (2, 44) = 7.362	P=0.0017		
		Time	1	F (1, 44) = 0.04728	P=0.8289		
	Accuracy of color test	Interaction	2	F (2, 44) = 0.1803	P=0.8356	SE = 0.9800 * LAE = 0.9703 * MAE = 0.9425 *	SE = 0.9787 LAE = 0.9467 MAE = 0.9205
		Group	2	F (2, 44) = 2.075	P=0.1377		
		Time	1	F (1, 44) = 0.8391	P=0.3646		
	Reaction time of color test	Interaction	2	F (2, 44) = 10.98	P=0.0001	SE = 1.170 LAE = 1.178 MAE = 0.9425	SE = 1.232 LAE = 1.184 MAE = 1.196
		Group	2	F (2, 44) = 17.44	P<0.0001		
		Time	1	F (1, 44) = 22.01	P<0.0001		
N-back	Accuracy of 1-back test	Interaction	2	F (2, 44) = 0.2563	P=0.7751	SE = 0.9728 LAE = 0.9771 MAE = 0.9612	SE = 0.9797 LAE = 0.9775 MAE = 0.9641
		Group	2	F (2, 44) = 0.3827	P=0.6842		
		Time	1	F (1, 44) = 0.8447	P=0.3631		
	Accuracy of	Interaction	2	F (2, 44) =	P=0.6986	SE =	SE =

	2-back test			0.3616		0.9466	0.9722
		Group	2	F (2, 44) = 1.054	P=0.3572	LAE = 0.9404	LAE = 0.9500
		Time	1	F (1, 44) = 5.568	P=0.0228	MAE = 0.9191	MAE = 0.9371

* Significant differences in SE *v.s.* MAE and LAE *v.s.* MAE pre-exercise, revealing no clinical effect.

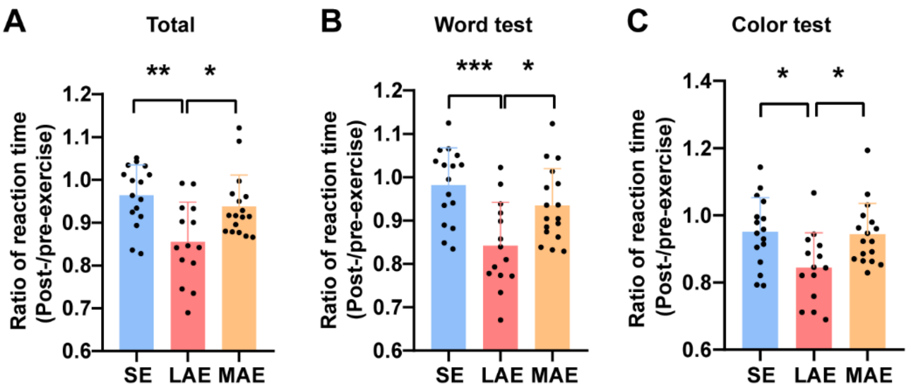

Fig. S1 Stroop task performance across different exercise groups

(A–C) the ratio of post- to pre-exercise reaction time in the Stroop task (A), Stroop word test (B) and color test (C). n = 16 for SE group, 14 for LAE group and 17 for MAE group, Brown-Forsythe Welch's ANOVA for accuracy and one-way ANOVA for reaction time.

* $p < 0.05$, ** $p < 0.01$, *** $p < 0.001$, **** $p < 0.000$, error bars indicate SD.

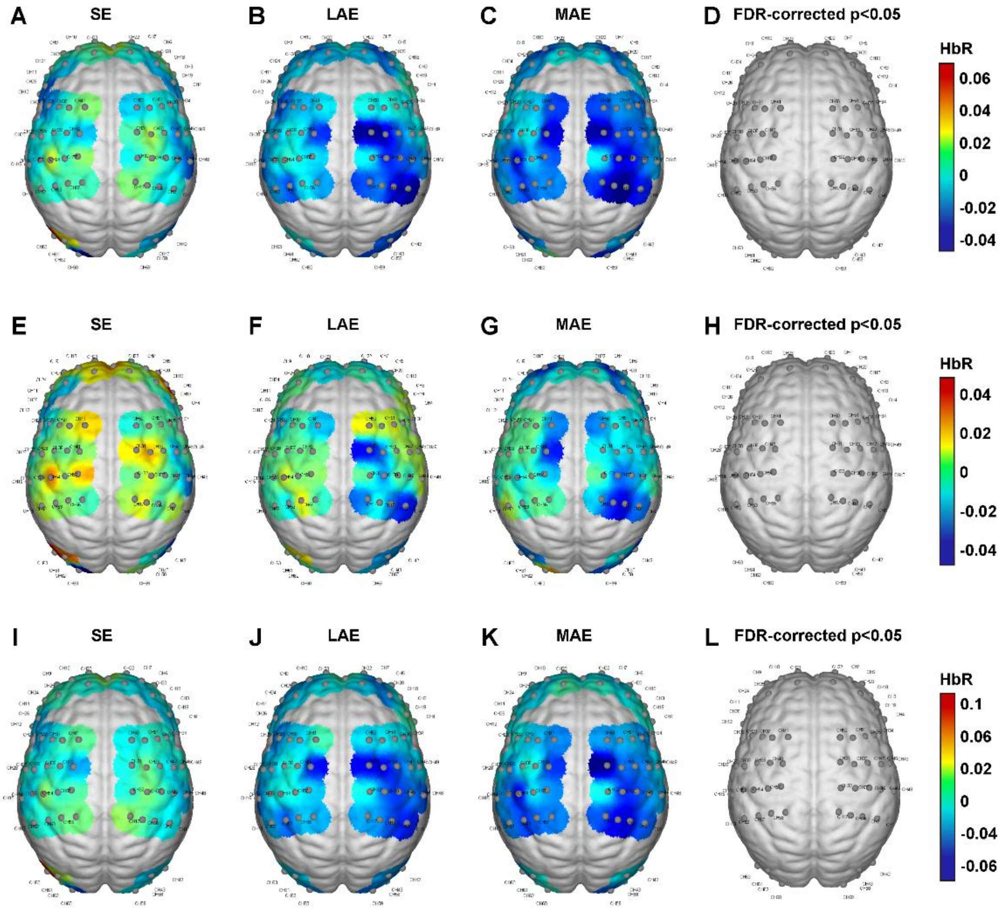

Fig. S2 fNIRS activation during Stroop task pre-exercise

(A–D) The Brain averaged activation maps from changes of HbR for SE group (A), LAE group (B) and MAE group (C) during Stroop task before exercise. And the statistically significant different channels (D) of the brain averaged activation maps from change of HbR among SE, LAE and MAE groups before exercise. n = 16 for SE group, 13 for LAE group and 17 for MAE group, one-way ANOVA with FDR correction applied.

(E–H) The Brain averaged activation maps from changes of HbR for SE group (E), LAE group (F) and MAE group (G) during the word test of Stroop task before exercise. And the statistically significant different channels (H) of the brain averaged activation maps from change of HbR among SE, LAE and

MAE groups before exercise. n = 16 for SE group, 13 for LAE group and 17 for MAE group, one-way ANOVA with FDR correction applied.

(I–L) The Brain averaged activation maps from changes of HbR for SE group (I), LAE group (J) and MAE group (K) during the color test of Stroop task before exercise. And the statistically significant different channels (L) of the brain averaged activation maps from change of HbR among SE, LAE and MAE groups before exercise. n = 16 for SE group, 13 for LAE group and 17 for MAE group, one-way ANOVA with FDR correction applied.

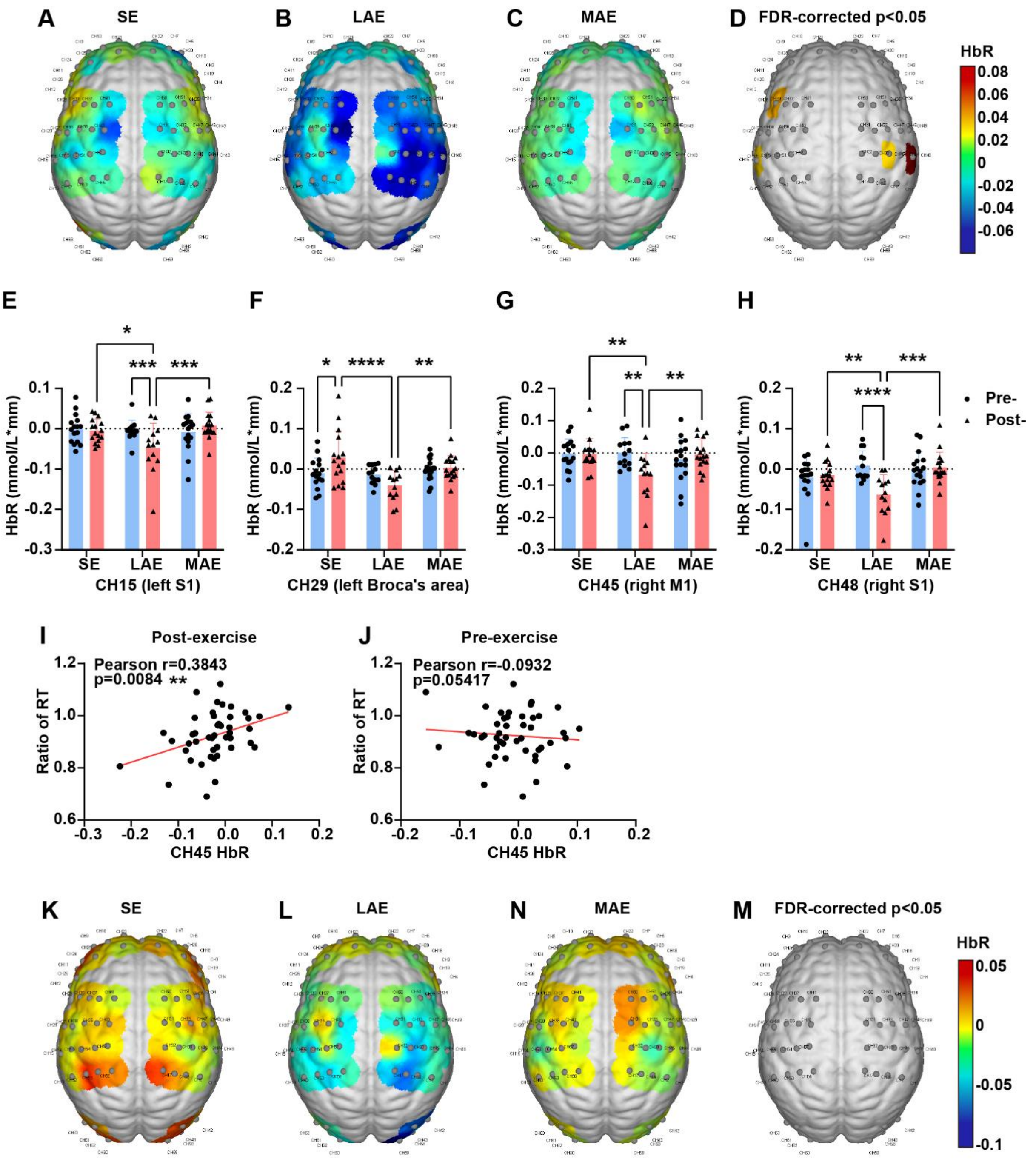

Fig. S3 fNIRS activation in word test and color test of Stroop task following exercise

(A–D) The Brain averaged activation maps from changes of HbR for SE group (A), LAE group (B) and MAE group (C) in word test of Stroop task post-exercise. And the statistically significant different channels (D) of the brain averaged activation maps from change of HbR among SE, LAE and MAE groups after exercise. n = 16 for SE group, 13 for LAE group and 17 for MAE group, one-way ANOVA with FDR correction applied.

(E–H) the HbR concentration in channel 15 (E), 29 (F), 45 (G) and 48 (H) among three groups pre- and post-exercise. n = 16 for SE group, 13 for LAE group and 17 for MAE group, two-way ANOVA.

(I–J) the relationship between the ratio of post- to pre-exercise reaction time of word test and the HbR concentration in channel 45 of three groups post- (I) and pre-exercise (J) among three groups. n = 16 for SE group, 13 for LAE group and 17 for MAE group, Pearson's correlation.

(K–M) The Brain averaged activation maps from changes of HbR for SE group (K), LAE group (L) and MAE group (N) in color test of Stroop task post-exercise. And the statistically significant different channels (M) of the brain averaged activation maps from change of HbR among SE, LAE and MAE groups after exercise. n = 16 for SE group, 13 for LAE group and 17 for MAE group, one-way ANOVA with FDR correction applied.

* $p < 0.05$, ** $p < 0.01$, *** $p < 0.001$, **** $p < 0.000$, error bars indicate SD.
